# Hypermethylation of the CHRDL1 promoter induces proliferation and metastasis by activating Akt and Erk in gastric cancer

**DOI:** 10.18632/oncotarget.15513

**Published:** 2017-02-19

**Authors:** Yao-fei Pei, Ya-jing Zhang, Yao Lei, Ding-wei Wu, Tong-hui Ma, Xi-qiang Liu

**Affiliations:** ^1^ Department of Hepatobiliary-Pancreatic Surgery, Zhejiang Provincial People's Hospital, Hangzhou, Zhejiang Province 310014, PR China; ^2^ Department of General Surgery, Bejing Anzhen Hospital, Capital Medical University, Beijing 100000, PR China; ^3^ Department of Interventional Therapy and Vascular Surgery, Hunan Provincial People's Hospital, Changsha, Hunan Province 410005, PR China; ^4^ Genetron Health (Beijing) Technology, Co. Ltd., Changping, Beijing 100000, PR China

**Keywords:** CHRDL1, EMT, gastric cancer, methylation, Wnt

## Abstract

CHRDL1 (Chordin-like 1) is a secreted protein that acts as an antagonist of bone morphogenetic protein (BMP). BMP plays a role as an activator of BMP receptor II (BMPR II), which mediates extracellular to intracellular signal transmission and is involved in carcinogenesis and metastasis. Herein, we report that CHRDL1 expression was significantly down-regulated in gastric cancer tissues and associated with poor survival. Clinic-pathological parameters demonstrated a close relationship between low CHRDL1 expression and metastasis. *In vitro*, CHRDL1 knockdown promoted tumor cell proliferation and migration through BMPR II by activating Akt, Erk and β-catenin. Furthermore, we observed the hypermethylation of the CHRDL1 promoter in gastric cancer, which induced low expression of CHRDL1 and decreased its secretion to the supernatant. Finally, *in vivo* experiments confirmed that CHRDL1 acted as a tumor suppressor gene in suppressing tumor growth and metastasis.

## INTRODUCTION

Gastric cancer is one of the most common malignant tumors globally [1]. Gastric cancer exhibits numerous characteristics, including invasion, aggression, metastasis and recurrence [2, 3]. Gastric cancer exhibits a high rate of recurrence and metastasis after surgical resection despite improvements in surgery and other therapy [4]. Therefore, it is necessary to further investigate the mechanism underlying tumorigenesis, metastasis and recurrence.

Bone morphogenetic protein (BMP) is a secretary protein that belongs to the transforming growth factor-β (TGF-β) super-family of growth factors. BMP signaling has attracted significant clinical interest because it is frequently involved in the dysregulation of cancer [5]. BMP binds to a BMP receptor (BMPR) on the cell surface and alters gene regulation through the phosphorylation of the Smad transcription factors [6]. The activation of intracellular signaling through BMPR can induce a series of responses, including proliferation, migration and invasion in many types of tumors [7].

It has been reported that BMPR is regulated by a range of extracellular components [8]. Many of these components are antagonists of BMP. These antagonists include noggin, CHRDL1 and twisted gastrulation (TSG) [9–11]. CHRDL1 is a specific antagonist of BMP. CHRDL1 inhibits the binding of BMP to BMPR through interaction with BMP4. CHRDL1 is secreted by the dorsal embryonic pole and contains a domain called the cysteine-rich von Willebrand factor type C (vWC), which can bind to BMP4 [12–14]. CHRDL1 plays an important role in embryonic cell differentiation and the adult brain [15, 16].

In our research, we observed that CHRDL1 expression was significantly down-regulated in gastric cancer and regulated by methylation status. Methylation-mediated gene silencing plays an important role in carcinogenesis [17, 18]. DNA methylation often affects CpG islands in gene promoters [19]. Many diseases are related to aberrant DNA methylation, such as gastric cancer, prostate cancer, breast cancer and hepatic cancer [20–23]. Hypermethylation status in the promoter region of tumor suppressor genes induces gene silencing and allows cancer cells to acquire enhanced proliferation, migration, invasion and anti-apoptotic potential [24–26]. The hypermethylation status of the CHRDL1 promoter observed in our study explained the low expression of CHRDL1 in gastric cancer tissues as compared with adjacent non-tumor tissues. Furthermore, the low expression of CHRDL1 led to its decreased secretion to the supernatant, induced BMPR activation and phosphorylation of Akt and Erk, and eventually increased Wnt-related gene expression. Our study is the first to clarify the methylation-mediated suppression of CHRDL1 expression and its contribution to the enhanced migratory potential of tumor cells.

## RESULTS

### CHRDL1 expression is down-regulated in gastric cancer and correlates with poor survival

**Figure 1:**
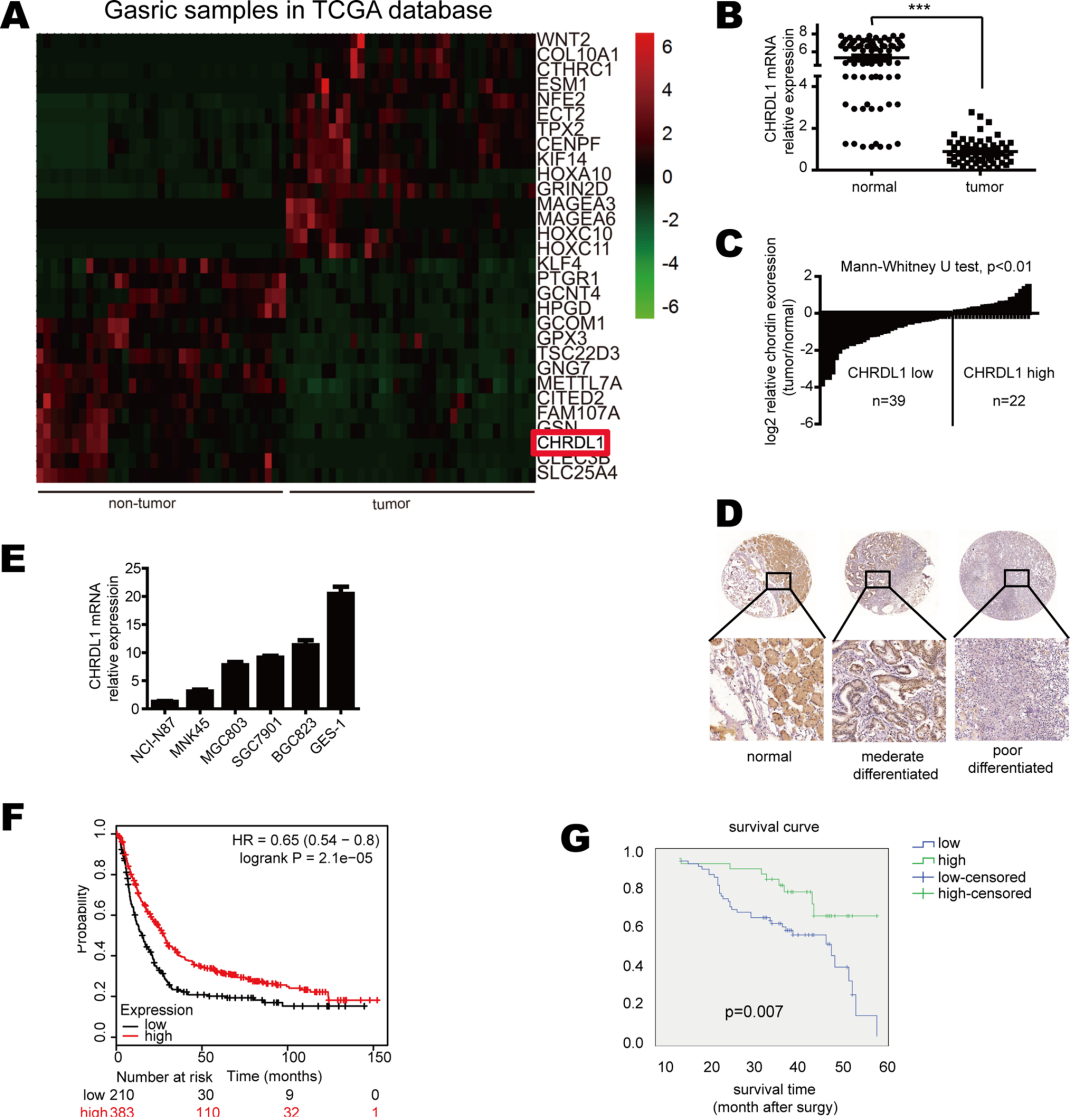
CHRDL1 expression was down-regulated in gastric cancer and correlated with poor survival (**A**) CHRDL1 expression was significantly down-regulated in tumor tissues compared with non-tumor tissues. (**B** and **C**) Data are presented as log_2_-fold changes by normalizing CHRDL1 expression in tumor tissues to non-tumor tissues. (**D**) IHC analysis of CHRDL1 in tumor tissues and non-tumor tissues. (**E**) CHRDL1 expression in gastric cancer cell lines. (**F**) Survival curve of patients with high or low CHRDL1 expression in TCGA and GEO database and (**G**) our clinical cohort (*n* = 100).

Autocrine protein production has been reported to be involved in carcinogenesis. BMP4 is an autocrine protein whose aberrant secretion can induce tumorigenesis, including gastric cancer. CHRDL1 is an antagonist of BMP4 and is also an autocrine protein. Although CHRDL1 has been reported to act as a tumor suppressor in breast cancer, there are few studies ofCHRDL1 in gastric cancer. To explore CHRDL1 expression in gastric cancer progression, we performed qPCR on 61 pairs of gastric cancer tissues and non-tumor tissues. Through analysis of TCGA database, we found CHRDL1 was significantly down-regulated in tumor tissues as compared with non-tumor tissues (Figure 1A). This result was further validated by qPCR with our clinical samples (Figure 1B and 1C). IHC also confirmed the down-regulation of CHRDL1 in gastric cancer tissues (Figure 1D). We also detected CHRDL1 expression in gastric cell lines and found that it was down-regulated in most gastric cancer cell lines, especially in NCI-N87 and MNK-45 compared with GSE-1 (Figure 1E).

We further analyzed the correlation between clinic-pathological parameters and CHRDL1 expression in gastric cancer patients. The patients were divided into two groups: high CHRDL1 expression group (*n* = 34) and low CHRDL1 expression group (*n* = 66). As shown in Table 1, low CHRDL1 expression had no correlation with age, gender, tumor size or differentiation. However, low CHRDL1 expression was significantly correlated with local invasion, lymph node metastasis and TNM stage (*p <* 0.05) (Table 1). The Kaplan-Meier plot of TCGA data base (Figure 1F) and our clinical samples (Figure 1G) both demonstrated that patients with low CHRDL1 expression exhibited poorer survival than those with high CHRDL1 expression, suggesting that CHRDL1 expression might act as a prognostic factor in patients with gastric cancer.

**Table 1:** Correlation between CHRDL1 expression levels and clinic-pathological parameters

Clinical-pathological parameters	CHRDL1 expression		*P* value
	Low (*n* = 66)	High (*n* = 34)	
Age (years) (missing case = 1)			0.447
≤ 60	33	14	
≥ 60	33	19	
Gender			0.118
Male	19	5	
Female	47	29	
Tumor size (cm)			0.374
≤ 5 cm	37	23	
≥ 5 cm	29	11	
Lauren classification (missing case = 1)			0.941
Intestinal	34	18	
Diffuse	32	15	
Differentiation			0.212
Well or moderately differentiation	19	14	
Poorly differentiation or undifferentiated	47	20	
Local invasion			0.001*
T1, T2	18	21	
T3, T4	48	13	
Lymph node metastasis			0.000*
No	20	24	
Yes	46	10	
TNM stage			0.002*
I, II	29	26	
III, IV	37	8	

### Low CHRDL1 expression promotes cell proliferation and migration through BMP4

**Figure 2:**
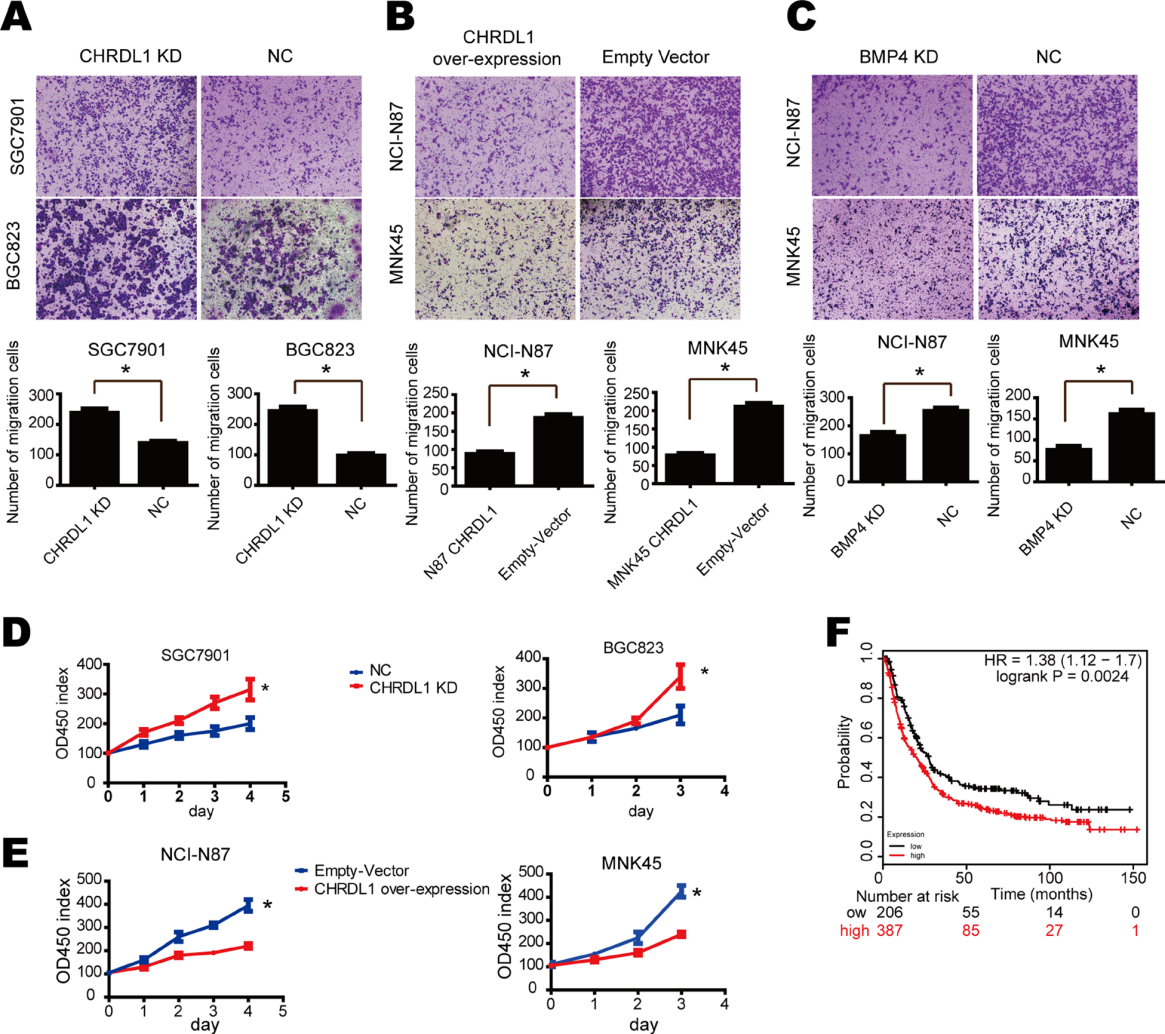
Low CHRDL1 expression promoted cell proliferation and migration through BMP4 (**A**) CHRDL1 knockdown in SGC7901 and BGC823 promoted cell migration. (**B**) CHRDL1 over expression in NCI-N87 and MNK45 inhibited cell migration. (**C**) BMP4 knockdown in NCI-N87 and MNK45 cells inhibited cell migration. (**D** and **E**) CHRDL1 knockdown promoted SGC7901 and BGC823 proliferation. CHRDL1 over expression inhibited proliferation of NCI-N87 and MNK45 cells. (**F**) Survival curve of patients with high or low BMP4 expression in TCGA database.

As shown in Table 1, low CHRDL1 expression correlated with metastasis. Therefore, we performed a transwell chamber assay to further confirm the influence of CHRDL1 on cell migration. As shown in Figure 2A, CHRDL1 knockdown by shRNA in SGC7901 and BGC823 cells enhanced cell migratory potential. However, when CHRDL1 was over-expressed in NCI-N87 and MNK-45 cells, cell migration was markedly inhibited (Figure 2B). Similar results were also observed in MGC803 cell, which had median expression of CHRDL1 (Supplementary Figure 1). These results suggested that CHRDL1 acted as a tumor suppressor gene. Next, we determined which pathway mediated the effects of CHRDL1 on gastric cancer. CHRDL1 has been reported to act as an antagonist of BMP4. BMP4 exists in the circulation and activates BMP receptor (BMPR II) to transmit extracellular signals to the cytoplasm. Thus, we knocked down BMP4 and observed decreased cell migration (Figure 2C). Furthermore, CHRDL1 knockdown promoted cell proliferation, while over expression inhibited cell growth (Figure 2D and 2E). And we also observed high expression of BMP4 indicated poor survival of gastric cancer patients in TCGA database (Figure 2F). Thus, low CHRDL1 expression promotes cell metastasis through BMP4 in gastric cancer.

### Low CHRDL1 expression actives Akt and Erk in gastric cancer

**Figure 3:**
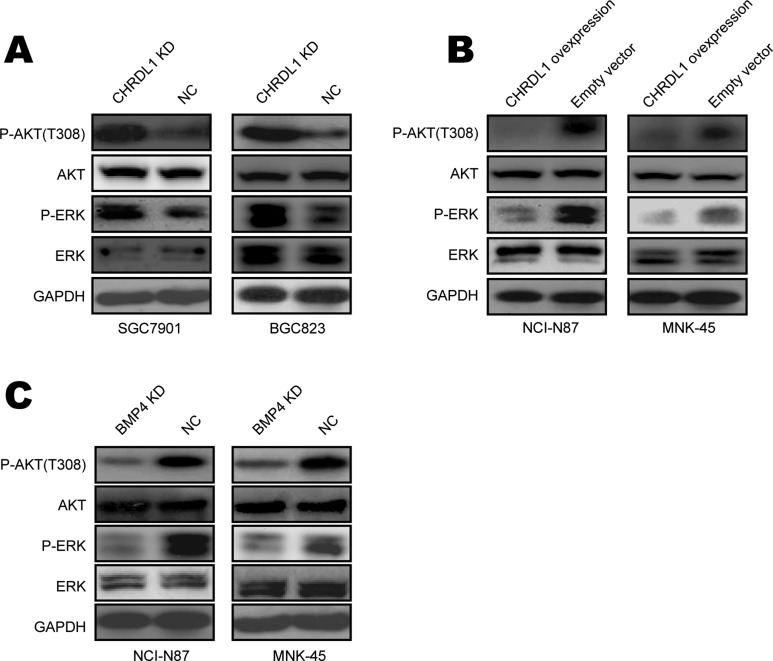
Low CHRDL1 expression activated Akt and Erk in gastric cancer (**A**) CHRDL1 knockdown in SGC7901 and BGC823 cells increased Akt and Erk phosphorylation. (**B**) CHRDL1 over expression in NCI-N87 and MNK45 cells decreased Akt and Erk phosphorylation. (**C**) BMPR II knockdown in NCI-N87 and MNK45 cells inhibited Akt and Erk phosphorylation.

To investigate the mechanism underlying how low CHRDL1 expression promotes cell proliferation and migration, we examined the levels of phosphorylated AKT, which is always involved in cell migration. As shown in Figure 3A, CHRDL1 knockdown increased Akt phosphorylation. Conversely, over expression of CHRDL1 induced Akt dephosphorylation (Figure 3B). Yang Y et al. reported that Akt and Erk can also be phosphorylated by BMPR II [27]. Therefore, we also measured Erk in our experiment. Erk phosphorylation was increased by CHRDL1 knockdown and decreased by over expression of CHRDL1 (Figure 3A and 3B). Because low CHRDL1 expression promoted cell proliferation and migration through BMP4, we knocked down BMP4 and observed decreased activation of Akt and Erk (Figure 3C). Collectively, these data demonstrate that low CHRDL1 expression activates Akt, Erk and induces cell proliferation and migration through BMP4 in gastric cancer.

### CHRDL1 knockdown activates the Wnt pathway

**Figure 4:**
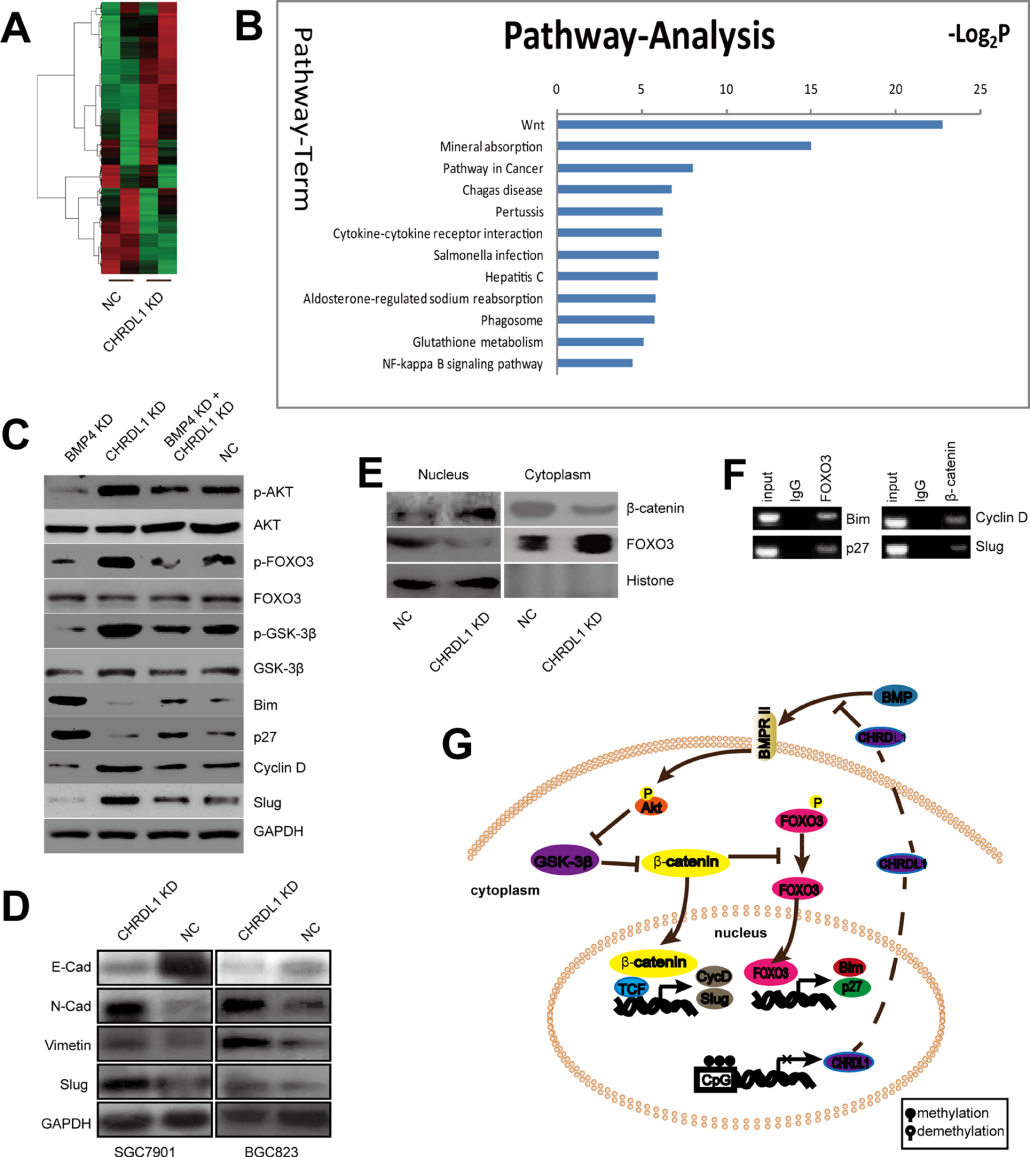
The Wnt pathway was activated by CHRDL1 knockdown (**A**) Heat map analysis of differentially expressed genes in the CHRDL1 KD and NC group with a cutoff value of 2/0.5. (**B**) Pathway enrichment of differentially expressed genes. (**C**) CHRDL1 knockdown decreased Bim and p27 expression but increased Cyclin D1 and Slug expression. (**D**) CHRDL1 knockdown induced EMT. (**E**) CHRDL1 knockdown promoted β-catenin translocation but inhibited FOXO3 nuclear translocation. (**F**) Ch-IP assay of β-catenin and FOXO3 binding to the target promoters. (**G**) Schematic illustration of the Wnt pathway was activated by CHRDL1 knockdown.

As illustrated above, down-regulation of CHRDL1 promoted cell proliferation and migration through activation of Akt and Epithelial-mesenchymal transition (EMT). However, the underlying mechanism remained unknown. A microarray was employed to identify the signaling pathway involved. BGC823 cells were divided into two groups: CHRDL1 knockdown (CHRDL1 KD) and NC group. As shown in Figure 4A, the candidate genes whose expression significantly differed between the two groups were selected using a cut-off value of 2/0.5. Pathway enrichment analysis indicated that the Wnt/β-catenin pathway was closely associated with CHRDL1 knockdown. The aberrant expression of genes involved in the Wnt signaling pathway was validated by western blotting (Figure 4B). As shown in Figure 4C, the β-catenin downstream genes Bim and p27 were down-regulated, whereas Cyclin D1 and Slug were up-regulated in the CHRDL1 KD group. The decreased Bim and p27 expression and increased Cyclin D1 expression induced cell proliferation. Increased Slug promoted migration through EMT induction, evidenced by substantial downregulation of E-Cad and upregulation of N-cad and Vimentin (Figure 4D). It has been reported that the β-catenin pathway is regulated by Akt/GSK3-β and FOXO3. Therefore, we also measured the GSK3-β and FOXO3 levels. CHRDL1 knockdown induced phosphorylation of Akt (Figure 3A) and GSK3-β (Figure 4C). Phosphorylated GSK3-β lost its ability to inhibit β-catenin. Consequently, the transcription of Cyclin D1 and Slug which were downstream of β-catenin was increased (Figure 4C). The activation of β-catenin also decreased dephosphorylated FOXO3 and its nuclear translocation (Figure 4C and 4E). So the transcription of FOXO3 target genes such as Bim and p27 were inhibited (Figure 4C). Chromatin immune-precipitation (Ch-IP) assay revealed that FOXO3 could directly bind to the promoters of Bim, p27 and that β-catenin could bind to the promoters of Slug and Cyclin D1 (Figure 4F).

Collectively, we demonstrated that low CHRDL1 expression activated Akt and Erk. Activated Akt stabilized β-catenin by phosphorylation of GSK3-β. Subsequently, β-catenin inhibited FOXO3 dephosphorylation and inhibited its nuclear translocation. As a result, Bim and p27 were down-regulated, whereas Cyclin D1 and Slug were increased, leading to tumor cell proliferation and metastasis (Figure 4G).

### Promoter hypermethylation down-regulates CHRDL1expression and secretion

**Figure 5:**
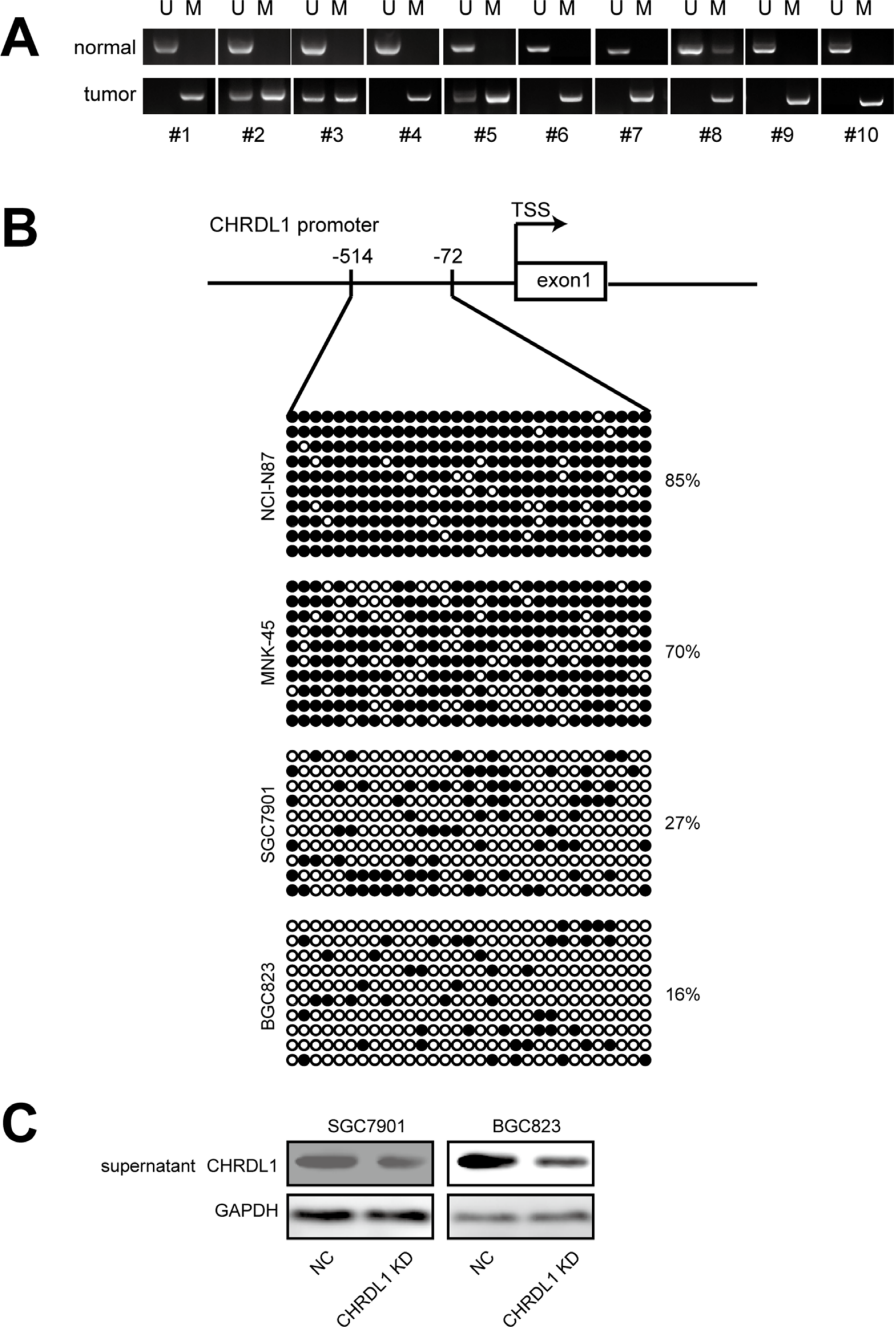
Hypermethylation in promoter down regulated CHRDL1 expression and secretion (**A**) The CHRDL1 promoter was heavily methylated in most tumor tissues compared with non-tumor tissues. (**B**) Methylation status of CHRDL1 in gastric cancer cell lines. (**C**) CHRDL1 knockdown decreased its levels in the supernatant.

Next, we investigated the underlying cause of low CHRDL1 expression in gastric cancer. As methylation-mediated gene silencing plays an important role in tumorigenesis, we measured the methylation status of the CHRDL1 promoter. First, methylation-specific PCR (MSP) was used to determine the methylation status of the CHRDL1 promoter in gastric cancer patients. As shown in Figure 5A, adjacent normal tissues exhibited promoter hypomethylation, whereas paired tumor tissues exhibited a hypermethylated status. To validate this result, we also examined gastric cancer cell lines used in our experiment. The CHRDL1 promoter was heavily methylated in NCI-N87 and MNK-45 cells, whereas hypomethylation was observed in SGC7901 and BGC823 cells (Figure 5B). The methylation status of the CHRDL1 promoter in these cell lines was consistent with the results shown in Figure 1D. As CHRDL1 inhibited cell migration through binding to BMP4 and blocking BMPR II signal transmission, we measured the levels of secreted CHRDL1 in the supernatant. As shown in Figure 5C, CHRDL1 knockdown decreased the CHRDL1 level in the supernatant. From these data, we concluded that the hypermethylation status of the CHRDL1 promoter in gastric cancer decreased CHRDL1 secretion in the supernatant, which promoted BMP binding to BMPR II and the subsequent activation of Akt and Erk.

### CHRDL1suppresses gastric cancer tumorigenesis and cell metastasis *in vivo*

**Figure 6:**
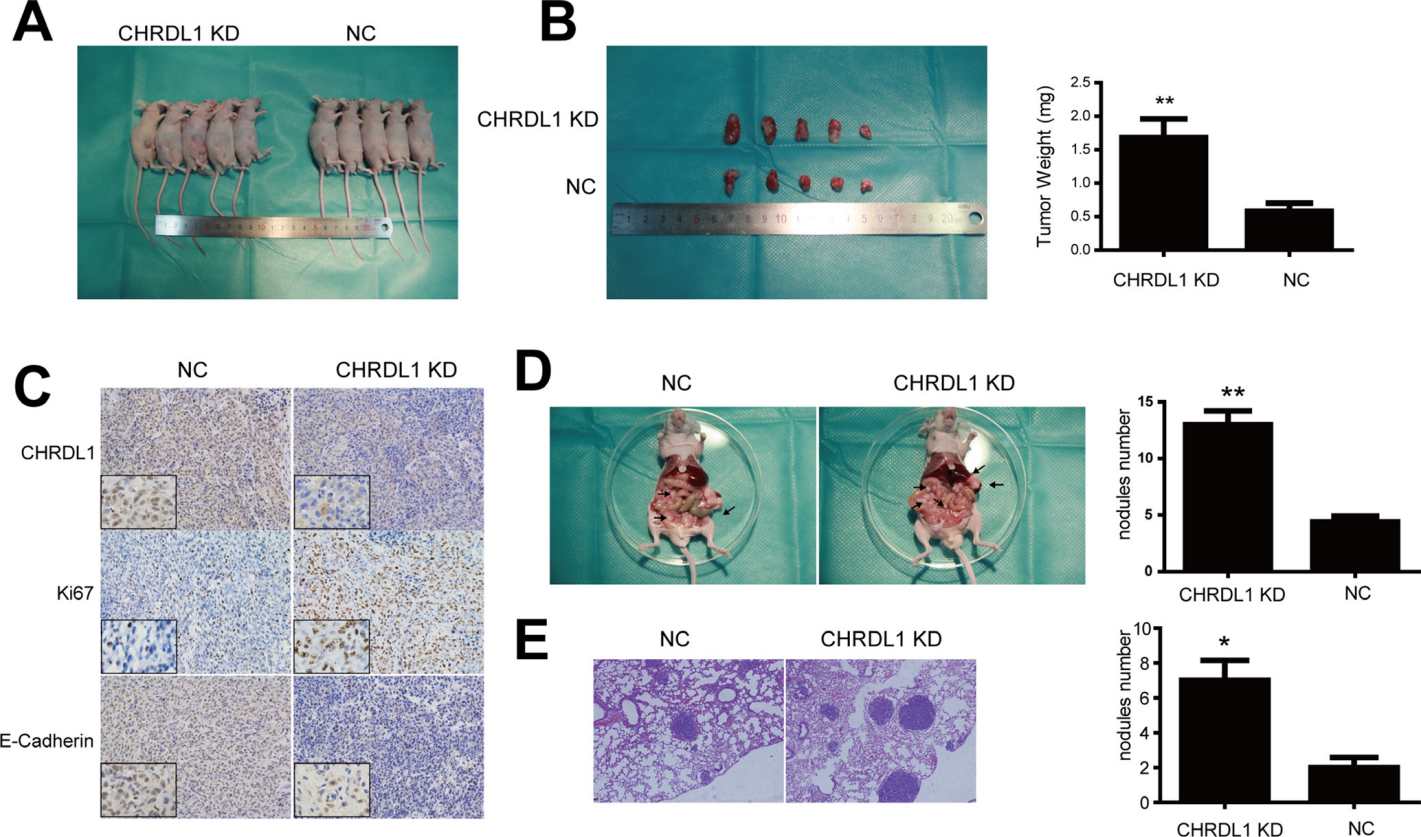
Xenograft model (**A** and **B**) CHRDL1 knockdown promoted tumor growth. (**C**) IHC analysis revealed that CHRDL1 knockdown induced higher Ki67 and lower E-Cadherin expression. (**D** and **E**) CHRDL1 knockdown promoted intraperitoneal and pulmonary metastasis.

Finally, we explored whether CHRDL1 could affect gastric cancer tumorigenesis and tumor cell metastasis *in vivo*. In this experiment, CHRDL1 was stably knocked down in BGC823 cells. CHRDL1 KD and NC cells were injected into nude mice subcutaneously, intraperitoneally and intravenously. After six weeks of injection, the tumor nodes in the CHRDL1 KD group were significantly larger than those in the NC group (Figure 6A and 6B). IHC analysis also revealed that the CHRDL1 KD group exhibited higher Ki67 and lower E-Cadherin than the NC group (Figure 6C). Moreover, there were fewer peritoneal and pulmonary metastatic nodules in the NC group thank in the CHRDL1 KD group (Figure 6D and 6E). Collectively, these data suggest that CHRDL1acts as a tumor suppressor gene and suppresses gastric cancer growth and metastasis.

## DISCUSSION

Secreted proteins in the circulation play an important role in carcinogenesis by binding to their receptors on the cell surface and transmitting extracellular signal to the intracellular milieu. The dysregulation of secreted proteins can induce a range of diseases, including malignancy [28]. Some secreted proteins can transmit positive signals that activate intracellular pathway, such as the Wnt signaling pathway, conferring an enhanced potential for proliferation, migration and invasion on tumor cells [29]. Other secreted proteins transmit negative signals that induce apoptosis or necrosis [30]. In our work, we determined that CHRDL1, a secreted protein, was markedly down-regulated in gastric cancer samples. Furthermore, we observed that CHRDL1 protein expression was closely correlated with tumor progression, including local invasion, lymph node metastasis and TNM stage. We also observed that patients with low expression of CHRDL1 exhibited poor survival.

Reduced CHRDL1expression led more BMP4 ligation to BMPRII and aberrant activation of the intracellular signal pathway members, such as Akt and Erk. Erk belongs to the MAPK family and its translocation into the nucleus to increase the transcription of genes that promote carcinogenesis. Aberrant activation of Erk can lead EMT, which promotes malignant transformation and enhances cell migration. Furthermore, the over expression of CHRDL1 increased the concentration of CHRDL1 in the supernatant and resulted in decreased Akt and Erk phosphorylation. The migration of gastric cancer cells was markedly inhibited by CHRDL1 over expression both *in vitro* and *in vivo*. To determine the underlying mechanism, we performed high-throughput pathway analysis and determined that genes downstream of the Wnt pathway were involved in this process. CHRDL1 knockdown activated Akt, promoted β-catenin nuclear translocation and activated the transcription of downstream genes that could induce cell proliferation and EMT. To investigate the mechanism by which CHRDL1was down-regulated in gastric cancer, we measured the promoter methylation status of CHRDL1 and observed hypermethylation in most gastric cancer samples relative to adjacent non-tumor tissues. These data suggest that promoter hypermethylation induces low CHRDL1 expression and subsequently activates BMPR II. BMPR II activation leads to the phosphorylation of Akt, Erk and β-catenin, eventually induces proliferation and EMT in gastric cancer cells and facilitates tumor cell metastasis. Therefore, CHRDL1 methylation status may represent a novel biomarker to identify and predict the prognosis of gastric cancer patients CHRDL1. Targeted therapy attracted more and more interest [31]. Our study of CHRDL1 may offer a new target for future treatment. However, our study is limited by the fact that we did not measure CHRDL1 levels in the serum of gastric cancer patients. Therefore, the relationship between serum CHRDL1 levels and the prognosis of gastric cancer patients requires further investigation.

Taken together, our data demonstrates that CHRDL1 acts as a tumor suppressor that is regulated by promoter DNA methylation. However, the underlying cause of CHRDL1 promoter hypermethylation remains to be determined. As epigenetic modulation is essential for cell differentiation and malignant cell transformation, the reason of CHRDL1 promoter hypermethylation will be studied in our future research.

## MATERIALS AND METHODS

### Gastric cancer samples and cell culture

A total of 100 pairs of tumor tissues and their adjacent non-tumor tissues were collected from gastric cancer patients who underwent surgery in Hunan Provincial People's Hospital, Changsha, China between 2011 and 2015. Samples from patients who underwent radiotherapy or chemotherapy were excluded. Tissues were stored at −80°C immediately. Written informed consents were obtained from patients. The study was approved by the Ethical Committee of Hunan Provincial People's Hospital and Zhejiang Provincial People's Hospital. All experiments were performed in accordance with the guidelines of the Ethics Committee of Hunan Provincial People's Hospital and Zhejiang Provincial People's Hospital. Gastric cancer cells NCI-N87, SGC-7901, BGC-823, MGC-803, AGS and MNK-45 were purchased from the American Type Culture Collection and cultured at 37°C with 5% CO_2_ in RPMI1640 medium containing 10% fetal bovine serum (Gibco).

### RNA extraction and qPCR analysis

Total RNA was extracted by Trizol Reagent according to the manufacturer's protocol (Invitrogen). RNA was reverse transcribed to cDNA using a reverse transcription kit (Promega, Madison, USA). qPCR was performed using the SYBR Green Mix (Takara, Tokyo, Japan). The primers used to detect CHRDL1 mRNA were as follows: CHRDL1 Forward- CCTGGAACC TTATGGGTTGGT, Reverse- AACATTTGGACATCTG ACTCGG; and GAPDH Forward-TTGGCATCGTTG AGGGTCT; Reverse-CAGTGGGAACACGGAAAGC. GAPDH was used as an internal control.

### CHRDL1 knockdown and over expression

The shRNA used to knock down CHRDL1 in gastric cancer cell lines was purchased from Shanghai GenePharma Co., Ltd. The shRNA sequences and negative control were synthesized as follows: CHRDL1 #sh1: ACGC CATGCACAGCATAATTT, #sh2 GTCCAAATGTTCA TTGCCTTT; BMP4 #sh1: TCCTTGAGGATAGACAGA, #sh2: GGGAGAAGCAGCCAAACTATG; NC: CCTAA GGTTAAGTCGCCCTCG. The shRNAs were packed into a lentivirus using pMD2G, pSPAX2 and shRNA2 according to the protocol provided by Clontech. The full-length human CHRDL1 cDNA was purchased from Addgene (Cambridge, MA, USA), subcloned into the pLVX-IRES-Puro vector, and packed into lentivirus with pMD2G and pSPAX2 to over express CHRDL1 in the target cells.

### Methylation specific PCR (MSP) and Bisulfite sequencing PCR (BSP)

Genomic DNA was extracted from tissues and gastric cell lines using a genomic DNA extraction kit and was treated by sodium bisulfate following the instructions of the Epi Kit (Qiagen, Germany). Sodium bisulfate-treated DNA samples were purified and amplified by PCR. Methyl Primer Express v1.0 software (ABI, Foster City, CA) was employed to design primers for BSP and MSP. The primers for MSP were as follows: CHRDL1 Forward: 5′- GAGTCGAGTAAGTCGTTGTTTTTTC-3′, Reverse: 5′- AAACTAAACACTAACCTTCTCCGTC-3′. Primers for BSP were as follows: CHRDL1 Forward: 5′- GGGAGTAGAAATAAAGTTATTGTGT-3′, Reverse: 5′- AAAAACTCAAAAATAAATAAAAAAAA-3′. PCR products amplified by BSP primers were ligated into the T-Vector (Takara, Tokyo, Japan). Ten clones of the T-Vector were randomly chosen and sent for sequencing. The methylation status was analyzed by an online tool (http://quma.cdb.riken.jp/).

### Western blotting

Western blotting was performed as previously described [32]. The primary antibodies against Akt, p-Akt (T308), Erk, p-Erk, E-Cadherin, N-Cadherin, Vimentin, Snail, and GAPDH and the HRP-conjugated secondary antibody were purchased from Cell Signaling Technology (MA, USA). The primary antibody CHRDL1 was purchased from Abcam (Cambridge, UK).

### Cell migration assay

For cell migration assays, cells were re-suspended in 200 μl serum-free medium and added into the upper chamber of transwell (Corning, NY, USA). In the lower chambers of the transwell, 600 μl medium containing 10% FBS was added. After 24 hr of culture, chambers were fixed and stained with 0.1% crystal violet. Cell numbers were counted using microscope (200× lens).

### Xenograft models

Six-week-old male BALB/c nude mice were purchased from the Shanghai SLAC Laboratory Animal Co., Ltd (Shanghai, China) and housed in a specific pathogen-free environment. BGC823 cells in which CHRDL1 was stably knocked down and their negative control cells were injected into mice subcutaneously, intraperitoneally and intravenously. Tumor volume was calculated as (W + L) /2 × W × L × 0.5236 (W: width, L: length) every 7 days. After four weeks, mice were euthanized. Pulmonary and peritoneal metastatic nodules were also calculated and expressed as the mean ± SD. Tumor grafts and pulmonary tissues were fixed, and IHC analysis was performed. All animal experiments were performed according to the guidelines of the Care and Use of Laboratory Animals of Zhejiang Provincial People's Hospital. All animal experiments were approved by the Ethical Committee of Zhejiang Provincial People's Hospital.

### Microarray and bioinformatics analyses

Total RNA was extracted from treated cells, and a human mRNA microarray chip (Agilent Technologies, USA) was used to analyze the differentially expressed mRNAs in BGC823 cells in which CHRDL1 was stably knocked down. Bioinformatics analysis was performed using GeneSping software (Agilent Technologies, USA) by JOINGENOME Co., Ltd. Hangzhou, China.

### Chromatin immune-precipitation

Chromatin immune-precipitation (Ch-IP) experiments were performed following the instructions within the chromatin immune-precipitation kit (Millipore). The primers to detect the promoter region were as follows: Bim Forward: 5′-TCGGACTGAGAAACGCAAGG-3′, Reverse: 5′-GGAAGCGGGCAGTAGGTGGT-3′; p27 Forward: 5′-TTGTTGGAGCAGTGAAATCTG-3′, Reverse: 5′-GG AACTCGTCCCTTTCTACTTT-3′; CyclinD Forward: 5′-TGCTGAAGGCGGAGGAGA-3′, Reverse: 5′-ATCCA GGTGGCGACGATC-3′; and Slug Forward: 5′-CGATGCC AACCTCCTCAA-3′, Reverse: 5′-ACGATCTTCCGCAT GGAC-3′.

### Statistical analysis

Statistical analysis was performed using SPSS 18.0 software (SPSS Inc., Chicago, IL, USA). The analysis of the relationship between CHRDL1 expression and clinic-pathological data was performed by Pearson χ^2^ test. Differences among groups were analyzed by Student's *t-test*. All experiments were repeated three times, and results are expressed as the mean ± SD. The results were considered statistically significant when *P <* 0.05.

## SUPPLEMENTARY MATERIALS FIGURE


