# Correction: Hypermethylation of the CHRDL1 promoter induces proliferation and metastasis by activating Akt and Erk in gastric cancer

**DOI:** 10.18632/oncotarget.19303

**Published:** 2017-07-17

**Authors:** Yao-fei Pei, Ya-jing Zhang, Yao Lei, Ding-wei Wu, Tong-hui Ma, Xi-qiang Liu

**Present:** The 4th author's name, ‘Ding-wei Wu,’ is spelled incorrectly.

**Correct:** The proper spelling is ‘Wei-ding Wu’.

Original article: Oncotarget. 2017; 8:23155-23166. https://doi.org/10.18632/oncotarget.15513

