# Supplementary material for: Hypermethylation of the CHRDL1 promoter induces proliferation and metastasis by activating Akt and Erk in gastric cancer
Source: Oncotarget. 2017 Feb 19;8(14):23155–66. doi: 10.18632/oncotarget.15513 (PMC5410293; doi:10.18632/oncotarget.15513)
Supplement: Supplementary file 1 [file oncotarget-08-23155-s001.pdf]

Hypermethylation of the CHRDL1 promoter induces proliferation and metastasis by activating Akt and Erk in gastric cancer

Supplementary Materials

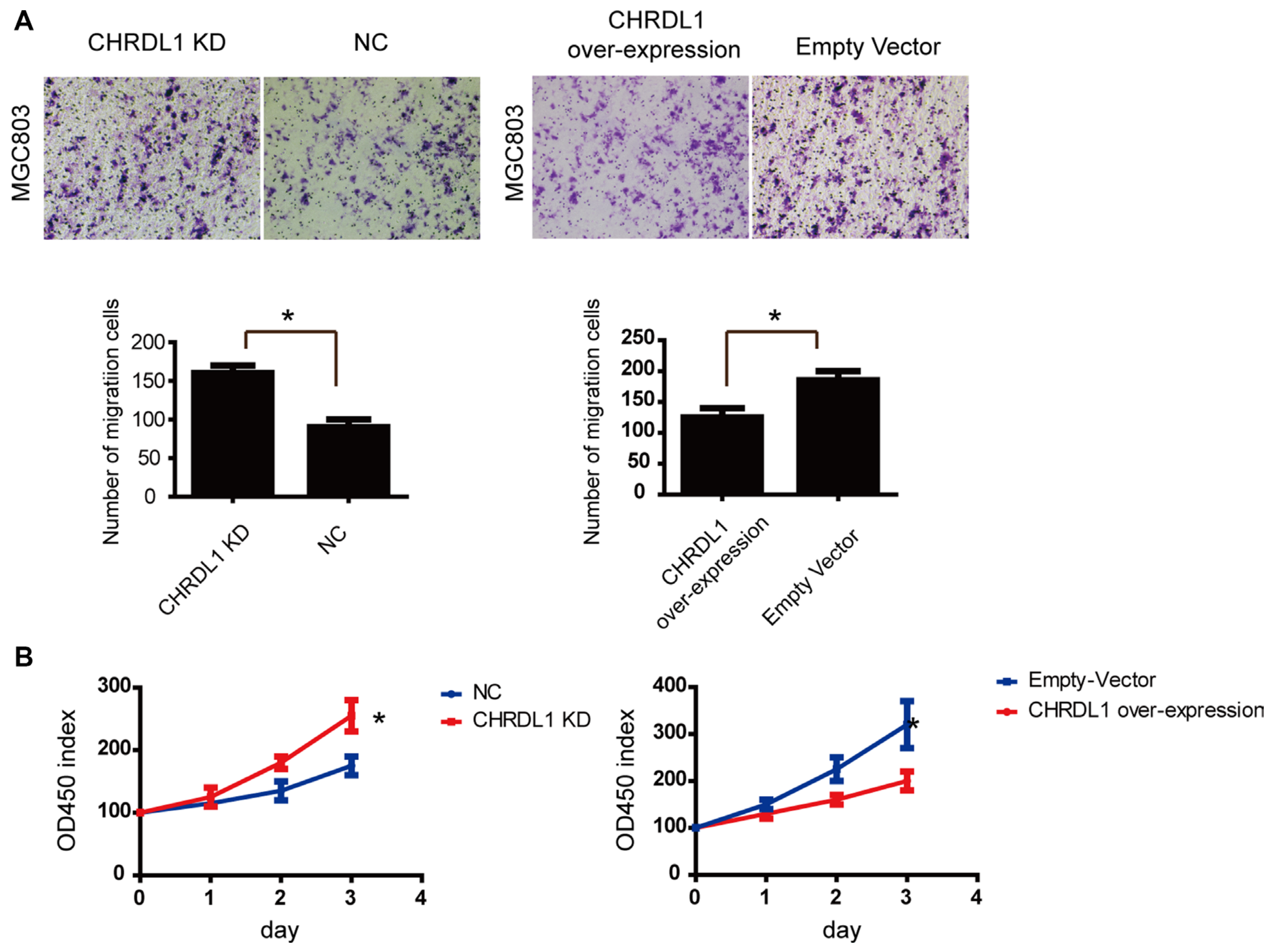

Supplementary Figure 1: Low CHRDL1 expression promoted cell proliferation and migration in MGC803. (**A**) CHRDL1 knockdown in MGC803 promoted cell migration, while CHRDL1 over-expression inhibited cell migration. (**B**) CHRDL1 knockdown in MGC803 promoted cell proliferation, while CHRDL1 over-expression inhibited cell proliferation.
